# Life-History Traits of the Black Soldier Fly, *Hermetia illucens* (L.) (Diptera: Stratiomyidae), Reared on Three Manure Types

**DOI:** 10.3390/ani9050281

**Published:** 2019-05-25

**Authors:** Chelsea D. Miranda, Jonathan A. Cammack, Jeffery K. Tomberlin

**Affiliations:** Department of Entomology, Texas A&M University, 2475 TAMU, College Station, TX 77845, USA; jcammack_07@tamu.edu (J.A.C.); jktomberlin@tamu.edu (J.K.T.)

**Keywords:** waste management, insect protein, confined animal facilities, sustainable agriculture, insect farming

## Abstract

**Simple Summary:**

The growing global human population raises concern about future food security. Such growth may lead to an increase in animal production, which raises concern about waste management. Rearing insects on animal manure could be an efficient solution to manage animal waste; but a better understanding of bioconversion of different manure types by targeted insects is necessary in order to develop such systems. Black soldier fly larvae are voracious feeders that are capable of digesting a wide range of organic material, including manure. Previous research has demonstrated that black soldier flies can convert manure into valuable biomass (i.e., aquaculture and poultry feed) while reducing nutrients and dry matter by 50% or more, and odorous volatile compounds by up to 100%. The purpose of this study was to evaluate the performance of select life-history traits of black soldier fly larvae fed three types of manure (poultry, swine, and bovine). The results may supplement existing data or give new perspective on how this insect may be used for waste management while producing an alternative feed.

**Abstract:**

Structural changes and growth of animal production systems have resulted in greater volumes of manure. Current manure storage methods pose a potential environmental threat. Lessening these issues is a key concern for the animal production industry. The primary aim of this research was to evaluate black soldier fly (BSF) performance when fed poultry, swine, or dairy manure at different rates (18 or 27 g/2 d until 40% prepupation). The results indicated that larvae fed with the control diet (Gainesville diet) were the heaviest (+31–70%); however, for other life-history traits, those fed the higher feed rate of poultry manure produced comparable results to the control. Larvae fed more resource, regardless of manure type, weighed more as larvae (+3–9%), pupae (+22–48%), and adults (+18–42%), developed faster (up to 3–4 d), had a higher percentage reach the prepupal stage (+2–16%), lived longer as adults (+1 d), and converted more resource to biomass (up to 1% more) than those fed at the lower rate. Yet, no difference was detected in dry matter (DM) reduction across feed rate for a given manure type. Based on these results, all three manure types can be digested by black soldier fly larvae, thus demonstrating their potential for waste management.

## 1. Introduction

The continued growth of the human population raises concerns regarding food security. Soymeal, fishmeal, and other protein sources may become limited, thus increasing the demand for alternative diet components for livestock and poultry [1,2]. Similarly, the recent increase in the number of confined animal facilities [3,4], due to changes in production costs, has resulted in large quantities of manure that must be properly managed. For example, in the United States (US) in 2012, factory-farmed livestock produced approximately 13 times the amount of sewage produced by the entire US population [5].

Currently, alternative methods for manure management are being sought to avoid issues with handling wastes. Manure from confined operations is typically stored in lagoons for long periods of time (6–12 months) [6], posing an environmental risk. Rearing insects on animal manure could be an efficient solution for managing wastes, while also producing protein, but a better understanding of the bioconversion of different manure types by targeted insects is necessary to develop efficient systems. 

One potential insect that could be utilized for waste management is the black soldier fly (BSF), *Hermetia illucens* (L.), (Diptera: Stratiomyidae) [7,8,9,10]. The black soldier fly is widely distributed in temperate and tropical regions throughout the world [11,12,13,14]. Often found near confined animal feeding facilities, BSF recycle manure, thus reducing associated nitrogen (N), phosphorus (P) and dry matter (DM) by 50% or more [7,9,15]. Additionally, BSF larvae convert poultry manure into a 35–44% protein [8,16,17] and 28–35% fat biomass (dry weight basis) [8,17]. Some suggest they are a suitable substitute for fishmeal for aquaculture production, such as catfish (*Ictalurus punctatus* Rafinesque) [18], rainbow trout (*Oncorhynchus mykiss* Walbaum) [19,20], blue tilapia (*Oreochromis aureus* (Steindachner)) [18], and turbot (*Psetta maxima* Linnaeus) [21]. Additionally, BSF can be incorporated into poultry [22], and swine [23] diets. The BSF is an ideal candidate for waste management because it is capable of reducing pathogens such as *Escherichia coli* [24] and *Salmonella* spp. [25], and is known to reduce offensive odors such as phenols, indoles, and volatile fatty acids [26]. Lastly, lipids can be extracted from the larvae for biofuel production [27], and the material remaining after larval feeding can be used as fertilizer [28], providing other potential applications of this species. However, one of the biggest obstacles BSF-fed-manure systems faces in the US is legislation. Currently, BSF are being sought out as alternative feed additives, but larvae fed manure are not permitted for food and feed purposes. Instead, BSF have been approved by the Association of American Feed Control Officials (AAFCO) for inclusion in aquaculture and poultry diets, but they must be fed ‘feed grade materials’ exclusively. This constraint for manure-fed BSF systems is due to the fact that manure is not regarded as safe as it is possible that larvae can bioaccumulate harmful compounds from manure. Charlton et al. [29] investigated the safety potential of BSF exposed to different contaminants via manure (e.g., veterinary medications, pesticides, heavy metals, mycotoxins, dioxins, polychlorinated biphenyls, and polybrominated diphenyl ethers) and found that all levels for all compounds in the larvae were below the European Union or Codex regulatory limits. However, this is the only known study to explore these concerns and it was for one population in Ghana; therefore, more research should be conducted before BSF fed manure are deemed safe and incorporated into animal diets. Despite the legal limitations for protein production from BSF fed manure in the US, they are utilized to manage manure and produce protein in other areas of the world, such as China (personal communication, Tomberlin).

The purpose of this study was to evaluate select life-history trait (from egg to adult) performance of BSF larvae fed three types of manure (poultry, swine, and bovine) as well as their ability to reduce dry matter (DM) and convert it into protein. As discussed previously, this species is capable of reducing manure, which may be an efficient solution to current management concerns. Furthermore, BSF can convert animal waste into a protein-rich biomass, which may also provide alternative means for protein production. Results will supplement existing data on how BSF may be utilized in waste management systems. 

## 2. Materials and Methods 

### 2.1. Acquisition of BSF

This experiment was conducted using BSF larvae from a colony (established in 2014) maintained at the Forensic Laboratory for Investigative Entomological Sciences (F.L.I.E.S.) Facility at Texas A&M University. This colony originated in 1998 from a colony at the Coastal Plain Experiment Station (University of Georgia) in Tifton, GA USA. The colony maintained at the F.L.I.E.S. Facility is reared according to methods detailed by Sheppard et al. [30].

### 2.2. Acquisition of Manure

Poultry manure less than 12-h-old was collected from layer hens housed at the Poultry Science Research, Teaching and Extension Center (Texas A&M University, College Station, TX, USA). Similarly, dairy manure was collected from a commercial dairy located in Stephenville, TX, USA, and swine manure from a local farm, in Anderson, TX, USA. Manure was collected before each trial. Once collected, manure was placed into 18.9 L buckets with lids (Home Depot^®^, Leaktite^TM^, Leominster, MA, USA) and transported to the F.L.I.E.S Facility, where it was homogenized (vigorously mixed by hand for approximately 5 min) and transferred to 3.76 L Ziploc^®^ Freezer Bags (S. C. Johnson & Son, Inc., Racine, WI, USA) and stored at −20°C until used. Manure was allowed to thaw at room temperature for 24 h before initiation of the experiment. Manure not used on day one was placed in 1.9 L Reditainer™ EXTREME FREEZE™ deli containers (Clear Lake Enterprises, Port Richey, FL, USA) and stored at 4 °C until used. Three 10 g samples of thawed manure of each type were used to determine initial moisture content gravimetrically [31].

#### Chemical Composition of Manure

Chemical composition of manure was analyzed at the Texas A&M AgriLife Extension Service Soil, Water, and Forage Testing Laboratory in College Station, TX, USA. Total N, is determined by a combustion process and mineral (B, Ca, Cu, Fe, K, Mg, Mn, Na, P, S, and Zn) are determined by an inductive coupled plasma spectrometry of a nitric acid digest. The chemical composition of the manures used in this study is detailed in Table 1.

### 2.3. Experiment Design

Adults were maintained in a 1.2 × 1.2 × 2.4 m wooden cage with each side of the cage lined with wire mesh in a greenhouse (25–30 °C, >50% relative humidity (RH)) to provide natural sunlight for mating and oviposition. Corrugated cardboard was cut into 4.0 × 4.0 × 0.5 cm pieces and three pieces of cardboard were taped together to form a bundle. Three bundles of corrugated cardboard were placed on the lid of 5.7 L Sterilite^®^ (Sterilite^®^ Corporation, Townsend, MA, USA) container with a 15 × 7 cm hole covered with wire cloth. The Sterilite^®^ container was filled with 1000 g Gainesville diet (50% wheat bran, 30% alfalfa meal, 20% corn meal) [32] saturated with reverse osmosis (RO) water. Every four hours the cardboard was checked for egg clutches. The cardboard containing eggs was dissected and egg clutches removed and placed in a 0.5 L plastic container, covered with a paper towel held in place with a rubber band, and stored in a Rheem Environmental Chamber (Asheville, NC, USA) at 29 °C, 60% RH, and 16L:8D until larvae eclosed. Newly-hatched larvae were fed 200 g of Gainesville diet as described by Sheppard, Tomberlin, Joyce, Kiser and Sumner [30] to decrease larval mortality prior to use in the experiment.

Replicates consisted of 100 4-d-old larvae placed in 88 mL Great Value^®^ Brand bathroom cups (Wal-Mart^®^ Stores, Inc., Bentonville, AR, USA) and provided manure as described below. Cups were covered with a paper towel held in place with a rubber band and stored in the growth chamber described above. As a control, 100 4-d-old larvae were placed on Gainesville diet (50% wheat bran, 30% alfalfa meal, 20% cornmeal) [32] and the methods described by Tomberlin et al. [33] were followed. Three replicates of each treatment and control were used and two trials were conducted.

Every other day, larvae in a treatment were fed their assigned manure type at a specified feed rate (18 g or 27 g) identified in preliminary experiments, or 27 g of Gainesville diet (10 g diet with 17 mL of water) [32]. Individual cups were each placed inside a 1.9 L Reditainer™ EXTREME FREEZE™ deli containers (Clear Lake Enterprises, Port Richey, FL, USA) covered with tulle fabric (Wal-Mart^®^ Stores, Inc., Bentonville, AR, USA) held in place with a rubber band. The containers were placed in a complete randomized block design in the incubator under the same conditions as previously described. The contents of the bathroom cups were dumped into the 1.9 L Reditainer™ EXTREME FREEZE™ deli containers (Clear Lake Enterprises, Port Richey, FL, USA) on day four due to concerns with moisture. Post-feeding larvae (identified in previously publications as prepupae) [8] were removed daily and feeding discontinued when 40% reached the prepupal stage as described by Tomberlin, Sheppard and Joyce [33]. 

In order to minimize the effect of handling, three of the largest larvae observed were selected from each replicate every three days, individually weighed on an OHaus^®^ Adventurer™ Pro AV64 balance (OHaus^®^ Corporation, Pine Brook, NJ, USA), and returned to their respective container. The mean larval weight (of three larvae) recorded on the day that 40% of the larvae within a treatment reach the prepupal stage was recorded as the final larval weight.

#### Immature and Adult Life-History Traits

Prepupae were removed daily from all treatments and weighed on the scale described above. Half of the prepupae produced were frozen at −20 °C. Those remaining were placed individually in 35 mL cups, covered with a plastic lid with a cotton ball inserted through a hole in the center of the lid, and returned to the growth chamber as a means to evaluate adult emergence time and associated longevity (monitored daily). Tomberlin, Sheppard and Joyce [33] suggested that individual adult flies provided with 0.125 mL of water lived longer than those not provided water. Therefore, the cotton ball was dampened daily with RO water and served as a source of water for the adults upon eclosion.

### 2.4. Bioconversion of Manure

To determine bioconversion, the total mass of all prepupae produced (wet weight) and amount of manure fed (wet weight) to each replicate over the course of the experiment were recorded. Percent bioconversion was calculated with:

(total g of all prepupae/total g of manure fed) × 100%

### 2.5. Percent DM Reduction

Percent DM reduction was determined as described by Zhou, Tomberlin, Zheng, Yu and Zhang [25]. The percent DM reduction was calculated with:

((W1 − W2)/W1) × 100%

with W1 being the DM provided during the experiment and W2 being the DM remaining at the end of the experiment. 

### 2.6. Protein Content of Larvae

To determine protein content of prepupae, samples were sent to SDK Laboratories in Hutchinson, KS, USA. Approximately 5 g of prepupae per replicate were needed for the protein analysis, which could not be obtained for the swine or dairy treatments. Therefore, statistical analyses were conducted only on prepupae fed poultry manure and Gainesville diet. 

### 2.7. Statistical Analysis

Moisture content of manure, life-history traits (final larval weight, development time from newly-hatched larvae to prepupal stage, percent prepupation, prepupal weight, adult male and female weight, adult male and female longevity), DM reduction, percent bioconversion, and protein content of prepupae, were analyzed across treatments. An ANOVA was performed for each parameter listed above using JMP 12.0.0 (SAS Institute Inc., Cary, NC, USA). Levine’s test for equal variances was used to check unequal variances and Tukey’s HSD (honest significant difference) was used for mean separation (*p* ≤ 0.05). 

## 3. Results

### 3.1. Moisture Content of Manure 

Initial moisture content differed significantly (F_3, 20_ = 296.1; *p* < 0.0001) across manure types. No significant treatment by trial interaction (F_1, 20_ = 0.1101; *p* = 0.9535) or trial effect (F_1, 20_ = 0.1278; *p* = 0.7241) were detected. Initial moisture content ranged from approximately 74–84%, with the highest found in dairy manure and lowest found in swine manure (Table 2).

### 3.2. Immature and Adult Life-History Traits

#### 3.2.1. Final Larval Weight

Final larval weight was significantly different (F_6, 28_ = 10.95; *p* < 0.0001) across larval diets and feed rates. No significant treatment by trial interaction (F_6, 28_ = 1.51; *p* = 0.2113) was found; however, a significant trial effect was (F_1, 28_ = 19.75; *p* < 0.0001). In general, individuals in trial one were 22% heavier than those in trial two. Furthermore, those provided Gainesville diet were the heaviest (0.20 g/larva) across trials (Table 3). With regards to treatment effect, those fed at the 18 g rate of poultry manure were the heaviest (0.14 g/larva). Those provide dairy or swine manure weighed 8% and 11% less than those provided the poultry manure, respectively. For those fed at the 27 g rate, the same pattern occurred. However, when comparing across feed rate for a manure type, larvae were in general 3–9% larger when provided more resource. 

#### 3.2.2. Time to first prepupation 

The time to the first observed prepupation was significantly different (F_6, 28_ = 62.27; *p* < 0.0001) across larval diets and feed rates. No significant treatment by trial interaction (F_6, 28_ = 1.58; *p* = 0.1906) was found. However, a significant trial effect was found (F_1, 28_ = 11.04; *p* = 0.0025). In general, individuals in trial two reached the prepupal stage 1 d faster than those in trial one. Furthermore, those provided Gainesville diet were the first to reach the prepupal stage, at 9 d after the experiment began (Table 3). With regards to treatment effect for those fed manure at the 18 g rate, larvae fed poultry manure reached the prepupal stage in 12 d. Those provide dairy or swine manure required 7 and 9 d longer to reach the prepupal stage than those provided the poultry manure, respectively. For those fed at the 27 g rate, the same pattern occurred. However, when comparing feed rates for a manure type, the larvae reached the prepupal stage up to 3–4 d faster when provided with more resource.

#### 3.2.3. Prepupal Weight 

Prepupal weight was significantly different (F_6, 28_ = 47.94; *p* < 0.0001) across larval diets and feed rates. No significant treatment by trial interaction (F_6, 28_ = 1.43; *p* = 0.2366) was found, but a significant trial effect (F_1, 28_ = 19.32; *p* < 0.0001) was. In general, individuals in trial one were approximately 13% larger than those in trial two. Furthermore, those provided Gainesville diet were the heaviest (0.13 g/larva) across trials (Table 3). With regards to treatment effect, those fed at the 18 g rate, larvae reared on poultry manure were the heaviest (0.09 g/larva). Those provided dairy or swine manure weighed 23% and 22% less than those provided the poultry manure, respectively. For those fed at the 27 g rate, the same pattern occurred. Still, when comparing across feed rate for a manure type, larvae fed higher feed amounts were 22–48% larger. 

#### 3.2.4. Percent Prepupation 

Percent pupation was significantly different (F_6, 28_ = 6.17; *p* = 0.0004) across larval diets and feed rates. No significant treatment by trial interaction (F_6, 28_ = 0.25; *p* = 0.9545) or trial effect (F_1, 28_ = 1.94; *p* = 0.1751) were found. With regards to treatment effect, larvae fed the Gainesville diet resulted in 86% pupation (Table 3), whereas for those fed manure at the 18 g rate, larvae reared on poultry manure resulted in the highest pupation percentage (94%), while those fed dairy (77%) or swine (70%) manure had fewer individuals reach the prepupal stage. A similar trend was observed for the 27 g rate. When comparing across feed rate for manure type, pupation in general was approximately 2–16% higher when larvae were provided more resource. 

#### 3.2.5. Adult Male Weight 

Adult male weight was significantly different across larval diets and feed rates (F_6, 28_ = 55.08; *p* < 0.0001). No significant treatment by trial interaction (F_6, 28_ =2.33; *p* < 0.0596) was found, but a significant (F_1, 28_ = 21.41; *p* < 0.0001) trial effect was. In general, individuals in trial one were approximately 14% heavier than those in trial two. Furthermore, those provided Gainesville diet were the heaviest (0.05 g/adult) (Table 3). With regards to treatment effect, for those fed at the 18 g rate, males fed poultry manure were the heaviest (0.04 g/adult), while those provide dairy or swine manure weighed 27% and 26% less, respectively. For those fed at the 27 g rate, the same pattern occurred. However, when comparing across feed rate for a manure type, males were in general 18–30% larger when provided more resource.

#### 3.2.6. Adult Male Longevity

Diet and feed rate had a significant impact on adult male longevity (F_6, 28_ = 21.92; *p* < 0.0001). No significant treatment by trial interaction (F_6, 28_ = 2.17; *p* = 0.0758) or trial effect (F_1, 28_ = 2.96; *p* = 0.0962) were found. Individuals provided Gainesville diet lived approximately 8 days (Table 3). With regard to treatment effect, for those fed at the 18 g rate, larvae fed poultry manure lived approximately 7 d, while those provided dairy or swine manure lived 29% and 32% less, respectfully. Similar results were observed for those fed the 27 g rate. However, when comparing across feed rate for a manure type, males lived in general 12–14% longer when provided more resource. 

#### 3.2.7. Adult Female Weight

Adult female weight was significantly different (F_6, 28_ = 27.47; *p* < 0.0001) across larval diets and feed rates. No significant treatment by trial interaction (F_6, 28_ = 2.11; *p* = 0.0837) was found. However, a significant (F_1, 28_ = 14.78; *p* = 0.0006) trial effect was found. In general, females in trial one were 15% heavier than those in trial two. Individuals provided Gainesville diet were the heaviest (0.07 g/adult) (Table 3). With regard to treatment effect, for those fed at the 18 g rate, larvae fed poultry manure were the heaviest (0.04 g/adult), while those provide dairy or swine manure weighed 21% and 24% less, respectively. The same pattern occurred for those fed the 27 g rate. Similarly, when comparing across feed rate for a manure type, females were in general 27–42% larger when provided more resource.

#### 3.2.8. Adult Female Longevity

Adult female longevity was significantly different (F_6, 28_ = 21.66; *p* < 0.0001) across larval diets and feed rates. No significant treatment by trial interaction (F_6, 28_ = 2.04; *p* = 0.0942) was found. Additionally, no significant (F_1, 28_ = 0.02; *p* = 0.8883) trial effect was found. Individuals provided Gainesville diet lived approximately 6.7 d. With regard to treatment effect (Table 3), for those fed at the 18 g rate, larvae fed poultry manure lived 6.5 d, while those provided dairy or swine manure lived 9% and 28% less, respectfully. For those fed at the 27 g rate, the same trend occurred. Though, when comparing across feed rate for a manure type, females lived in general 2–11% longer when provided more resource.

### 3.3. Percent DM reduction. 

**Figure 1:**
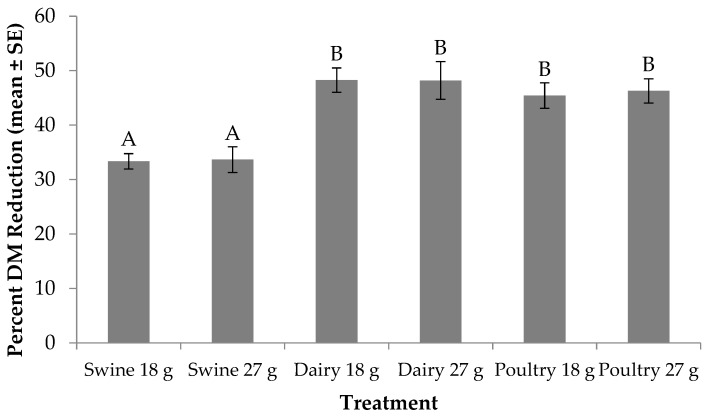
Percent dry matter (DM) reduction (mean ± SE, ^1^*n* = 6) for black soldier fly larvae fed swine, dairy or poultry manure at two feed rates every other day at 29 °C, 60% RH, and 16L:8D. Different letters indicate significant differences between treatments (α = 0.05), ANOVA followed by Tukey’s HSD (honest significant difference). ^1^*n* = number of replicates per treatment.

Percent DM reduction was significantly different across larval diets and feed rates (F_5, 24_ = 7.78; *p* = 0.0002). No significant treatment by trial interaction (F_5, 24_ = 0.06; *p* = 0.9971) or trial effect (F_1, 24_ = 2.96; *p* = 0.0984) was found. With regard to treatment effect, for those fed the 18 g rate, larvae provided dairy manure resulted in the highest DM reduction (48%), while those provided poultry and swine reduced the DM by 2% and 15% less, respectively (Figure 1). A similar trend occurred for those fed the 27 g rate. Yet, when comparing across feed rate, individuals fed less dairy or swine manure reduced DM by up to 2% more; however, more DM was reduced for those fed the higher feed rate of poultry manure. 

### 3.4. Percent Bioconversion 

**Figure 2:**
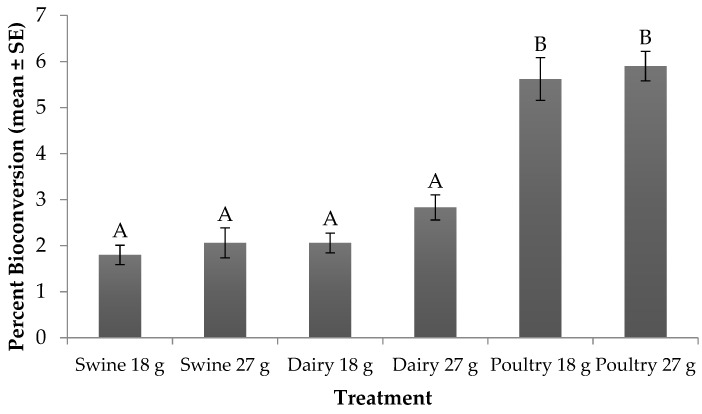
Percent bioconversion (mean ± SE, ^1^*n* = 6) for black soldier fly larvae fed swine, dairy or poultry manure at two feed rates every other day at 29 °C, 60% RH, and 16L:8D. Different letters indicate significant differences between treatments (α = 0.05), ANOVA followed by Tukey’s HSD (honest significant difference). ^1^*n* = number of replicates per treatment.

Significant differences (F_5, 24_ = 44.23; *p* < 0.0001) in percent bioconversion were found across larval diets and feed rates. No significant treatment by trial interaction (F_5, 24_ = 2.34; *p* = 0.0727) or trial effect (F_1, 24_ = 1.25; *p* = 0.2742) were found. With regard to treatment effect, for those fed the 18 g rate, larvae provided poultry manure resulted in the highest bioconversion (5.6%), while those fed swine and dairy converted 1.8% and 2.1% of the manure into biomass, respectively (Figure 2). A similar trend occurred for those fed the 27 g rate. Yet, when comparing across feed rate for a manure type, larvae fed more manure converted up to 1% more resource to biomass than those fed less.

### 3.5. Protein Content of Prepupae

**Figure 3:**
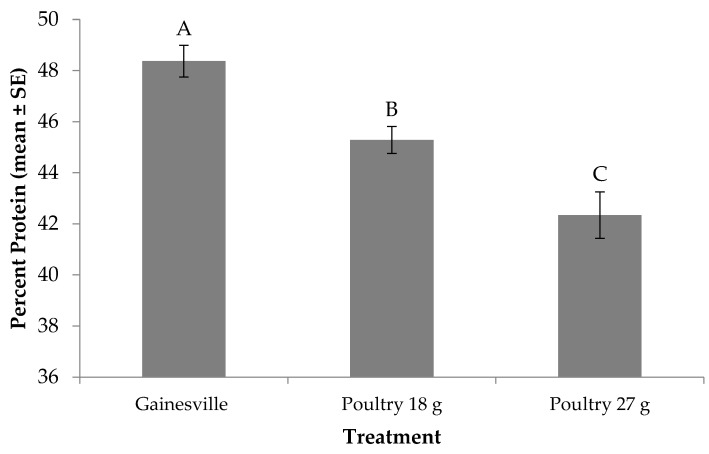
Percent protein (mean ± SE, ^1^*n* = 6) for black soldier fly larvae fed Gainesville diet [32] or poultry manure at two feed rates every other day at 29 °C, 60% RH, and 16L:8D. Different letters indicate significant differences between treatments (α = 0.05), ANOVA followed by Tukey’s HSD (honest significant difference). ^1^*n* = number of replicates per treatment.

The protein content of prepupae was significantly affected by diet (F_2, 12_ = 18.70; *p* = 0.0002). No significant treatment by trial interaction (F_2, 12_ = 1.41; *p* = 0.2827) or trial effect (F_1, 12_ = 0.57; *p* = 0.4657) were found. Prepupae produced from the Gainesville diet contained approximately 48% protein, while those produced from the 18 or 27 g of poultry treatments contained approximately 3 points and 6 points less, respectively (Figure 3). When comparing across feed rate for poultry manure, prepupae produced on the lower feed rate had higher protein content than those fed more manure. 

## 4. Discussion

The results from this study indicate manure type and feed rate significantly influence the development of BSF. Regardless of feed rate, larvae fed poultry manure weighed more as prepupae (15–37%), developed faster (4–9 d), had a higher percentage reach the prepupal stage (3–24%), lived longer as adults (1–3 d) and converted more resource to biomass (3–4%) than those fed dairy or swine manure (Table 3). Furthermore, when comparing individuals within a manure type, larvae fed the higher feed rate weighed more as larvae (3–9%), pupae (22–48%), and adults (18–42%), developed faster (up to 3–4 d), had a higher percentage reach the prepupal stage (2–16%), lived longer as adults (1 d), and converted more resource to biomass (up to 1% more) than those fed at the lower rate. However, no differences were detected across feed rate for a given manure type for DM reduction. These data are necessary in optimizing biomass production and waste management practices.

There are only two known studies that concurrently compared the development of BSF on different manure types [9,16]. Results from the current study agree with those reported by Zhou, Tomberlin, Zheng, Yu and Zhang [16], who found that larvae of three different BSF strains (one from Texas and two from China) fed poultry manure, weighed on average 17–28% and 134–208% more than those fed swine and dairy manure, respectively. Specifically, larvae that originated from the Texas strain fed poultry manure weighed 28% and 208% more than those fed swine and dairy manure, respectively. Larvae from the current study fed poultry manure weighed 9–12% and 15–30% more than those fed swine or dairy manure, respectively. However, larvae from the previous study were dried, whereas our results are reported on a wet-weight basis. Additionally, in the previous study, 300 6-d-old larvae were fed ad libitum, which differs from the age of larvae initially inoculated into the manure, larval density, and feeding regimen used in the current study. Still, a similar trend occurred when comparing the studies demonstrating that those fed poultry manure weighed more than those fed swine or dairy manure. In a similar study, Oonincx, van Huis and van Loon [9] placed 100 newly-hatched larvae on poultry, swine, and dairy manure, that had been previously dehydrated and then rehydrated to 66% moisture. This study focused on different life-history traits than the study by Zhou, Tomberlin, Zheng, Yu and Zhang [16]: survivorship and development time to the first prepupae. They found that larvae fed swine manure resulted in higher larval survivorship (97%) compared to those fed poultry (82%) and dairy (88%) manures. Although Oonincx, van Huis and van Loon [9] measured larval survivorship to the first prepupa was detected and our study measured total number to successfully reach the prepupal stage, results from the current study do not agree with those reported by Oonincx, van Huis and van Loon [9] as larvae fed poultry manure resulted in higher percent prepupation (94–95%) compared to those fed swine (70–84%) and dairy (77–93%) (Table 3). Additionally, Oonincx, van Huis and van Loon [9] found that the development time for those fed swine manure was not significantly different from than to those fed poultry manure, which was not the case in our study (Table 3); larvae reared on swine manure took 5–8 days more than when reared on poultry manure. Differences in the results between the current study and Oonincx, van Huis and van Loon [9] could be due to differences in the chemical composition of the manures tested or methods employed. The N content in the chicken manure used by Oonincx, van Huis and van Loon [9] was higher compared to the current study (4.8 vs 2.1%), but was similar for swine and dairy. However, for P, manure used in the current study was higher for poultry manure (2.4 vs 1.2%). Also, they dehydrated and then rehydrated the manures, which likely reduced the microorganisms and significantly extended the BSF development to 144–215 d. It is known that microorganisms impact BSF development [34] and moisture impacts microbial growth [35]; therefore, drying the manure may have influenced the results reported by Oonincx, van Huis and van Loon [9]. However, if BSF fed manure are intended to be used as a feed additive, a pre-treatment of the manure, such as drying seems necessary to circumvent safety concerns. Future research should explore pre-treatment options for BSF fed manure. 

Past research has indicated nutritional composition of the diet can impact immature [36] and adult life-history traits [37]. Black soldier flies are able to develop in various types of wastes, which makes them ideal candidates to manage multiple waste streams. The larvae are considered generalist feeders capable of digesting a wide range of organic wastes [38]. However, when compared to other substrates, such as poultry feed, kitchen wastes, liver, and fish renderings, BSF fed swine manure consumed the resource at a slower rate [39] and weighed 8–40% less [38]. These results are expected as manure contains less energy and nutrients than the other waste types [38,39]. 

Different types of manure have different chemical contents (Table 1), and as such, variations in observed life-history traits of flies fed different manure types are expected. For example, poultry manure is typically higher in protein, amino acids, and minerals and lower in fiber than dairy manure [40,41]. Furthermore, these manures vary in the ratio of nutrients and moisture [41], which is known to impact BSF development. Black soldier flies fed a balanced diet of protein and carbohydrates (21:21, each at 21% of the diet) developed faster and consumed less compared to those fed protein- (35:7) or carbohydrate- (7:35) rich diets [36]. Additionally, moisture content of the substrate influences BSF development [36,42], and this may have also impacted our results, as initial moisture content for the manures used in this study varied by 3–10% (Table 2). Larval nutrition is important, especially for a species like *H. illucens* that rely on their fat bodies acquired during larval development to sustain their adult livelihood. For example, Gobbi, Martínez-Sánchez and Rojo [37] showed that larval diet influenced adult wing size and ovarian development in BSF, providing evidence that larval nutrition can impact population dynamics. Still, inconclusive results have been reported for the effects of different larval diets on other adult traits such as longevity and egg production [36]. This is important because past research on other flies has suggested that longevity and egg production may be inversely related [43] and it has been postulated that they may be influenced by the duration of emergence time [36] or delays in mating [33] as resources are being reallocated to sustain livelihood, rather than directed towards reproductive purposes. In the current study, larvae fed poultry manure resulted in heavier adults with increased longevity (Table 3). As such, future studies should investigate differences in ovipositional preference for those fed manure to optimize production systems.

The amount of resource provided to larvae can influence life-history traits. Myers, Tomberlin, Lambert and Kattes [7] fed 300 4-d-old larvae 27, 40, 54, or 70 g of dairy manure daily. For the purposes of comparing the aforementioned results with ours, we will focus on those fed 27 g and 40 g daily, as these feed rates are most similar to ours. Results from the study by Myers, Tomberlin, Lambert and Kattes [7] indicate that feeding more resource (40 vs. 27 g/d) decreased development time by 6%, increased prepupal weight by 33%, increased adult weight by 35% and longevity by 15%; however, 22% more DM was reduced by feeding less resource. Our results show larvae fed more dairy manure also have shorter development times (by 3 d), are larger as prepupae (by 48%) and adults (by 23–29%) and live longer as adults (by 2–12%). Albeit, those fed more dairy manure reduced less DM in our study, the difference across feed rates was not as great as reported by Myers, Tomberlin, Lambert and Kattes [7] and may be considered negligible (0.12% difference). Other differences between Myers, Tomberlin, Lambert and Kattes [7] and our study may be due to differences in the density (100 vs. 300 larvae) and feeding frequency (feeding daily vs. every other day). Additionally, different results may be expected in systems that utilize bulk-feeding regimes as Banks et al. [44] showed that 100 larvae fed one bulk feeding (of human feces) develop slower but weigh 19% more than those fed incrementally, but there was no significant difference in waste reduction capabilities between the two feeding regimes. However, significant differences (*p* ≤ 0.05) were detected for waste reduction for other densities tested (1 and 10 larvae/treatment) with higher reductions occurring in incremental systems. These findings show that feeding regimen is relevant in terms optimizing biomass production or waste management as incremental systems may extend development by one day compared to batch feeding systems [45]. Nevertheless, higher biomass is desired, feeding one bulk feeding [44] or more resource (in incremental systems) [7] is recommended. However, for waste management purposes, increased DM reduction is achieved in incremental systems [44] with less resource [7].

From an industrial perspective, co-digestion of different wastes may be an alternative method to increase larval production efficiency on lower quality substrates. In the current study, larvae fed dairy manure reduced 2–15% more DM than those fed poultry or swine manure (Figure 1). However, more resource was converted to larval biomass for those fed poultry manure (Figure 2). Dry matter and bioconversion were not measured for the control diet in this study, which was an oversight. Such data should be included in future studies with similar questions being addressed. Still, co-digestion of dairy manure with poultry manure offers a way to manage multiple waste streams while increasing DM reduction and bioconversion, as well as increasing survivorship and decreasing development time [10]. A similar trend for increased DM reduction, bioconversion, and survivorship, along with decreased development time was observed when dairy manure was co-digested with soybean curd [46]. Likewise, mixing wastes offers the opportunity to manipulate the nutrient content of the larvae. For example, fish offal can be mixed with dairy manure to increase larval biomass as well as enrich larvae with omega-3 fatty acids [47]. Such systems that involve co-digestion should be further investigated to improve BSF production and waste management systems.

Sheppard, Newton, Thompson and Savage [8] suggested utilizing BSF for on-farm poultry manure management. This species is an attractive means to manage animal waste because they can break down cellulose, lignocellulose, and hemicellulose [10]. Additionally, they deter house fly, *Musca domestica* L. (Diptera: Muscidae) oviposition [48], reduce odorous volatile compounds by up to 100% [26] and manure accumulation and nutrients by 50% or more [7,9,15], and recycle it into a protein-rich biomass [8]. In this study, larvae fed the Gainesville diet had a higher percent protein (48%) than those fed poultry manure; yet, when comparing across feed rate for those fed poultry manure, those fed the lower feed rate had a higher percent protein (45%) compared to those fed the higher feed rate (42%) Although not tested in the current study, differences in percent protein for those fed different manure types is expected, as Zhou, Tomberlin, Zheng, Yu and Zhang [16] showed that protein content of larvae from three different BSF strains fed poultry, swine or dairy manure varied; specifically, those fed poultry manure had higher protein contents than those fed the other two manure types. Past research has also suggested that larval diet influences protein [49] and lipid accumulation [50], and as previously discussed, ultimately influences adult life-history traits [36], which in turn, impacts population dynamics. Therefore, in order to utilize these insects for waste management or food and feed purposes, a better understanding of their life history parameters is necessary to develop efficient systems. 

## 5. Conclusions

In this study, the results show significant differences in weight, development and survivorship across the manure types tested. In all cases, BSF could be used to recycle these wastes and produce protein. However, these results are based on a bench-top experiment and may not easily translate on a larger production scale. Future research should examine the development of BSF fed different manures under mass rearing conditions. 

## Figures and Tables

**Table 1:** Chemical composition (mean ± SE) of swine, dairy, and poultry manure for the black soldier fly experiment.

%	Swine	Dairy	Poultry
N	2.10 ± 0.02 ^A^	2.10 ± 0.03 ^A^	2.40 ± 0.04 ^B^
P	2.42 ± 0.08 ^A^	0.66 ± 0.11 ^B^	2.43 ± 0.05 ^A^
K	0.01 ± 0.02 ^A^	0.57 ± 0.00 ^B^	2.44 ± 0.01 ^C^
Ca	4.36 ± 0.05 ^A^	1.96 ± 0.19 ^B^	14.0 ± 0.47 ^C^
Mg	0.87 ± 0.03 ^A^	0.67 ± 0.00 ^B^	0.56 ± 0.00 ^B^
Na	0.55 ± 0.01 ^A^	0.32 ± 0.02 ^B^	0.58 ± 0.00 ^A^
Zn	0.08 ± 0.00 ^A^	0.02 ± 0.00 ^B^	0.04 ± 0.00 ^C^
Fe	0.01 ± 0.00 ^A^	0.27 ± 0.04 ^B^	0.19 ± 0.02 ^B^
Cu	0.01 ± 0.00 ^A^	0.00 ± 0.00 ^B^	0.00 ± 0.00 ^B^
Mn	0.05 ± 0.00 ^A^	0.02± 0.00 ^B^	0.05 ± 0.00 ^A^
S	0.78 ± 0.01 ^A^	0.41 ± 0.02 ^B^	0.86 ± 0.00 ^C^
B	0.08 ± 0.00 ^A^	0.00 ± 0.00 ^B^	0.00 ± 0.00 ^B^

Different letters within a row indicate significant differences between treatments (α = 0.05), ANOVA followed by Tukey’s HSD (honest significant difference).

**Table 2:** Initial moisture contents (mean ± SE) of swine, dairy, and poultry manure, and Gainesville [32] for the black soldier fly experiment.

Manure Type	Feed Amount (g/2 days)	Initial (%)
Swine	18	73.94 ± 0.41 ^A^
	27	
Dairy	18	83.92 ± 0.63 ^B^
	27	
Poultry	18	77.24 ± 0.29 ^C^
	27	
Gainesville	27	70.00 ± 0.56 ^D^

Different letters within a column indicate significant differences between treatments (α = 0.05), ANOVA followed by Tukey’s HSD (honest significant difference).

**Table 3:** Comparison of life-history traits (mean ± SE, ^1^*n* = 6) of black soldier fly larvae fed swine, dairy, or poultry manure at two feed rates or Gainesville diet [32] every other day at 29 °C, 60% RH, and 16L:8D.

Treatment		Final Larval Weight (g)	Time to First Prepupation (d)	Prepupal Weight (g)	Percent Prepupation	Adult Male Weight (g)	Adult Male Longevity (d)	Adult Female Weight (g)	Adult Female Longevity (d)
Swine	18	0.1264 ± 0.0063 ^A^	20.3 ± 1.1 ^A^	0.0678 ± 0.0029 ^A^	69.5 ± 5. 3 ^A^	0.0273 ± 0.0016 ^A^	4.9 ± 0.3 ^A^	0.0320 ± 0.0018^A^	4.7 ± 0.1 ^A^
	27	0.1189 ± 0.0132 ^A^	16.7 ± 0.6 ^B,C^	0.0827 ± 0.0009 ^A,B^	83.8 ± 6.0 ^A,B,C^	0.0321 ± 0.0019 ^B^	5.5 ± 0.2 ^A^	0.0405 ± 0.0028 ^B,C^	5.1 ± 0.2 ^A,B^
Dairy	18	0.1306 ± 0.0093 ^A^	18.6 ± 0.7 ^A,B^	0.0668 ± 0.0016 ^A^	76.7 ± 3.4 ^A,B^	0.0271 ± 0.0004 ^A^	5.1 ± 0.2 ^A^	0.0330 ± 0.0009 ^B,C^	5.9 ± 0.2 ^A^
	27	0.1339 ± 0.0118 ^A^	15.6 ± 0.6 ^C^	0.0990 ± 0.0054 ^B,C^	92.7 ± 3.1 ^B,C^	0.0334 ± 0.0017 ^B,C^	5.7 ± 0.4 ^A^	0.0427 ± 0.0023 ^B^	6.0 ± 0.2 ^B,C^
Poultry	18	0.1419 ± 0.0089 ^A^	11.6 ± 0.2 ^D^	0.0866 ± 0.0016 ^A,B^	93.7 ± 2.7 ^B,C^	0.0371 ± 0.0012 ^C^	7.2 ± 0.2 ^B^	0.0420 ± 0.0019 ^B,C^	6.5 ± 0.2 ^C,D^
	27	0.1541 ± 0.0168 ^A^	11.3 ± 0.3 ^D,E^	0.1137 ± 0.0085 ^C,D^	95.3 ± 1.7 ^C^	0.0483 ± 0.0022 ^D^	8.2 ± 0.3 ^C^	0.0598 ± 0.0044 ^C^	7.2 ± 0.3 ^D^
Gainesville ^2^	27	0.2019 ± 0.0020 ^B^	9.0 ± 0.0 ^E^	0.1324 ± 0.0008 ^D^	86.0 ± 2.9 ^A,B,C^	0.0516 ± 0.0014 ^D^	8.1 ± 0.1 ^B,C^	0.0653 ± 0.0014 ^C^	6.7 ± 0.3 ^C,D^

Different letters within a column indicate significant differences between treatments (α = 0.05), ANOVA followed by Tukey’s HSD (honest significant difference). ^1^*n* = number of replicates per treatment. ^2^ Larvae were fed the standard diet (10 g of dry Gainesville diet + 17 mL of water) following the methods described by Tomberlin, Sheppard and Joyce [33].
